# Self-Assembly of an Organized Cementum-Periodontal Ligament-Like Complex Using Scaffold-Free Tissue Engineering

**DOI:** 10.3389/fphys.2019.00422

**Published:** 2019-04-11

**Authors:** Avik Basu, Kristi Rothermund, Meer N. Ahmed, Fatima N. Syed-Picard

**Affiliations:** ^1^ Department of Oral Biology and Center for Craniofacial Regeneration, School of Dental Medicine, University of Pittsburgh, Pittsburgh, PA, United States; ^2^ Department of Bioengineering, Swanson School of Engineering, University of Pittsburgh, Pittsburgh, PA, United States; ^3^ McGowan Institute for Regenerative Medicine, Pittsburgh, PA, United States

**Keywords:** periodontal ligament stem cells, periodontium, tissue engineering, self-assembly, organoid, periodontal regeneration

## Abstract

A major challenge in regenerating periodontal tissues is emulating its complex structure containing both mineralized and soft tissues. In this study, scaffold-free tissue constructs engineered using periodontal ligament cells (PDLCs), which contain a population of adult stem/progenitor cells, self-assembled into an organized multi-tissue structure comprising a mineralized cementum-like core enclosed within a periodontal ligament (PDL)-like tissue. Scaffold-free engineered constructs were formed by culturing human PDLCs to form a cell sheet on six-well dishes containing two minutien pins placed 7 mm apart. The cell sheet was contracted by the cells to roll into the pins forming a cylindrical construct anchored on either end by the pins. These tissues were approximately 1 mm in diameter and 7 mm long and contained only the cells and their endogenous matrix. These scaffold-free engineered constructs exhibited two structurally distinct tissues, one in the center of the construct and another on the periphery. The center tissue was mineralized and expressed alkaline phosphatase and bone sialoprotein, similar to cementum. The peripheral tissue was not calcified and expressed periodontal ligament-associated protein-1 and periostin, which is characteristic of the periodontal ligament. This tissue organization was seen after *in vitro* culture and maintained *in vivo* following subcutaneous implantation in immunocompromised mice. These data demonstrate that scaffold-free tissue engineering facilitates PDLCs to self-assemble into an organized cementum-PDL-like complex. These engineered tissues could be used as implantable grafts to regenerate damaged periodontal tissues or as model systems to study PDLC biology and mechanisms driving organized tissue assembly within the periodontium.

## Introduction

The periodontium is composed of multiple specialized tissues surrounding the tooth root that function to support the tooth and anchor it to the jaw. The root surface is lined by mineralized cementum, which is tethered to the alveolar bone by the periodontal ligament (PDL), and all of these structures are covered by the gingiva. It is estimated that 38.5% of adults in the United States have moderate-to-severe periodontitis (Eke et al., 2012), a disease that results in the breakdown of the cementum, PDL, and alveolar bone and leads to tooth loss. Developing a successful and predictable therapy to repair these structures is therefore of great importance. Rebuilding a complex structure like the periodontium comprising both soft and hard tissues is a major challenge in regenerative medicine. The achievement of this goal will require several concurrent processes including the recruitment and differentiation of cells to form multiple tissues in a spatially organized manner (Bartold et al., 2016). A better understanding of the biology of the cells involved in these processes and the molecular signals driving cell differentiation and tissue patterning within the periodontium would lead to the development of enhanced therapies to treat periodontitis.

A population of stem/progenitor cells has been identified in the periodontal ligament that are clonogenic and multipotent with the ability to differentiate into cementoblasts, PDL fibroblasts, and osteoblasts (Seo et al., 2004). A significant amount of research is directed toward developing new cell-based therapies to treat periodontitis using these periodontal ligament cells (PDLCs). However, creating these types of therapies involves stimulating PDLCs to regenerate the multiple periodontal tissues in an anatomically correct manner and preventing disorganized or spontaneous tissue formation that could lead to anomalies such as ankyloses. To address this challenge, many researchers are designing complex scaffolds with graded properties to direct the formation of organized periodontal tissues (Sowmya et al., 2017; Varoni et al., 2018; Wu et al., 2018). During development, cells drive the synthesis and assembly of organized multi-tissue structures using their endogenous extracellular matrix (ECM) for structure. The optimal approach of generating an effective therapy involves understanding and emulating these developmental processes.

Scaffold-free tissue engineering is a method of tissue regeneration where cells generate and organize their endogenous ECM to form a three-dimensional (3D) structure. Unlike traditional tissue engineering methods, scaffold-free tissue engineering bypasses the use of an exogenous scaffold material to form a 3D tissue. Because of this, scaffold-free tissue engineering methods follow similar pathways of tissue assembly seen naturally during development. A number of scaffold-free tissue engineered constructs have been shown to be able to self-assemble into organized multi-tissue or organ constructs (Muraglia et al., 2003; Bosnakovski et al., 2004; Syed-Picard et al., 2009, 2013, 2014; Smietana et al., 2010; Eiraku et al., 2011; Lee et al., 2017; Ovsianikov et al., 2018). Previously, we have shown that scaffold-free tissues formed from bone marrow stromal cells organize into a bone-like structure covered with an outer periosteum, and scaffold-free dental pulp cell constructs organize into a dentin-pulp complex (Syed-Picard et al., 2009, 2013, 2014; Smietana et al., 2010). In this study, we found that similar 3D, scaffold-free constructs generated from PDLCs self-assemble into a spatially organized PDL-cementum-like complex. We have characterized cell differentiation and tissue assembly within these engineered tissues after *in vitro* culture and *in vivo* implantation. The development of an engineered tissue that organizes into multiple structures of the periodontium could be used as a graft tissue to replace structures damaged by periodontitis and would also provide a powerful model system to study PDLC biology and periodontal tissue assembly yielding important mechanistic information that will enhance future cellular therapies to treat periodontitis.

## Materials and Methods

### Periodontal Ligament Cell Isolation

Healthy human third molars were obtained from the University of Pittsburgh School of Dental Medicine following routine extraction. The collection of human third molars for these studies was not considered human subject research because the teeth were discarded tissues and were completely de-identified prior to being transferred to the investigators of this study. Therefore, patient consent was not required to use this tissue. Teeth were collected from both male and female patients within the age range of 12–22 years. The teeth were cleaned in a solution of phosphate buffered saline containing penicillin-streptomycin (P/S; Gibco), and the PDL was dissected from the root surface of the teeth. The PDL tissue was minced with a scalpel and digested in an enzyme solution containing 3 mg/ml collagenase (MP Biochemical) and 4 mg/ml of dispase (Worthington Biochemical) at 37°C for 30–60 min. The digest was then passed through a 70-μm cell strainer to obtain a single cell solution. Cells were plated at an initial density of 10–20,000 cells/cm^2^ in growth medium (GM) containing Dulbecco’s Modified Eagle Medium (DMEM; Gibco), 20% fetal bovine serum (FBS; Atlanta Biologicals), and 1X P/S. Cells were expanded and cryogenically frozen for future experiments. Cells were used for experiments between passages 2 and 4. Cells isolated from different patients were kept separate, not pooled. All *in vitro* experiments were repeated using cells from at least three different patients to ensure reproducibility.

### Formation of Scaffold-Free Engineered Tissues

Scaffold-free constructs were formed using similar methods as previously described (Syed-Picard et al., 2009, 2013). Construct dishes were prepared by filling wells of six-well plates with 3 ml of polydimethylsiloxane (PDMS; Dow Corning), and the PDMS was allowed to cure to form a solid layer at the bottom of the wells. The PDMS was coated with 3 μg/cm^2^ laminin (Gibco) to facilitate PDLC adhesion, and the dishes were exposed to UV light for approximately 90 min. PDLCs were plated onto these construct dishes at an initial density of 200,000 cells/well in GM supplemented with 5 mg/ml ascorbic acid (Sigma), 10^−8^ M dexamethasone (dex; Sigma), 5 mM β-glycerophosphate (βGP; MP Biochemical), and 5 ng/ml fibroblast growth factor 2 (FGF2; Peprotech). Once the cells became confluent, 2 minutien pins (Fine Science Tools) were positioned in the center of the dish approximately 7 mm apart, and the culture medium was switched to now containing DMEM with 5% FBS, 5 mg/ml ascorbic acid, 10^−8^ M dex, 5 mM βGP, 5 ng/ml FGF2, and 2 ng/ml transforming growth factor β1 (TGFβ1; Peprotech). Cells formed tissue sheets that naturally detached from the substrate and contracted toward the pins to form a cylindrical scaffold-free tissue, anchored on either end by the pins. Constructs were cultured for an additional 7 or 14 days after formation of *in vitro* analyses or constructs were implanted subcutaneously in mice after 7 days in culture. Dexamethasone, ascorbic acid, and βGP are medium supplements known to induce osteogenic differentiation. FGF2 and TGFβ1 were also added to our culture medium in order to fully emulate the culture conditions used to previously form these types of scaffold-free engineered tissues from dental pulp cells and bone marrow stromal cells (Syed-Picard et al., 2009, 2013). This facilitated the ability to make direct comparisons across engineered tissues formed from cells originating from different tissues.

To ensure the biological and technical reproducibility of the results presented in this study, we have engineered scaffold-free PDLC constructs using cells isolated from a total of five different patients. In total, for these studies, we have generated 80 (*n* = 80) scaffold-free PDLC constructs.

### Animal Implantation

All animal procedures were approved by the University of Pittsburgh Institute of Animal Care and Use Committee. Balb/c immunocompromised mice, 8–10 weeks in age, were anesthetized through the inhalation of isoflurane gas, and the surgical field was cleaned with betadine and ethanol. Approximately 1 cm incisions were made through the skin on the backs of the mice, and a subcutaneous pocket was created through blunt dissection. One scaffold-free PDLC construct was placed into each pocket, and four samples were placed into each animal. A total of 16 scaffold-free PDLC constructs were implanted into animals for this study; we performed experiments using four animals, implanting four samples/animal.

### Histology and Immunofluorescent Staining

Following *in vitro* culture or *in vivo* implantations, samples were fixed in a solution of 4% paraformaldehyde overnight. Subsequent to fixation, some samples were decalcified in a solution of 0.4 M ethylenediaminetetraacetic acid (EDTA) for 1–2 h. Samples were embedded in paraffin, and 5 μm longitudinal sections were prepared. Sections were stained for hematoxylin and eosin (H&E) to characterize cell and tissue morphology and structure, and un-decalcified sections were also stained for Alizarin Red to detect mineralization. Immunostaining was performed to detect bone sialoprotein (BSP; Millipore); periostin (Novus); periodontal ligament-associated protein 1 (PLAP-1), also known as asporin (Invitrogen); or alkaline phosphatase (ALP; Abcam). Fluorescently tagged secondary antibodies, Alexa Fluor 546 goat anti-mouse and Alexa Fluor 488 goat anti-rabbit (Invitrogen), were used to detect the signal. Negative controls to our immunostaining were performed in parallel by omitting the primary antibody. DAPI (4′,6-diamidino-2-phenylindole, dihydrochloride) staining was performed to detect nuclei, and slides were mounted with Immu-mount aqueous mounting medium (Thermo Scientific). Brightfield images were acquired using a Nikon Eclipse TE200-E inverted microscope, and fluorescent images were taken using a Nikon Eclipse Ti inverted microscope.

### Micro-Computed Tomography (μCT)

μCT scans of the engineered tissues were acquired both after *in vitro* culture and following *in vivo* implantation to detect and localize mineral deposition. The tissues were fixed in 4% paraformaldehyde overnight. The samples were then dried overnight in air. μCT scans were taken using a Scanco μCT 50 system (Scanco Medical AG, Bruttisellen, Switzerland). The scans were performed in air with the following parameters: 45 kVp energy, 10 μm voxel size, 200 μA intensity, 0.36° rotation step (180° angular range), 800 ms exposure, and 1 average frame per view. The Scanco μCT software (HP, Open VMS/DECwindows Motif 1.6) was used for 3D reconstruction and viewing of images. A threshold of 550–1,000 units (gray scale) was set to visualize mineral and 180–550 units (gray scale) to visual soft tissue.

## Results

### Characterization of Scaffold-Free PDLC Constructs Following *in vitro* Culture

**Figure 1:**
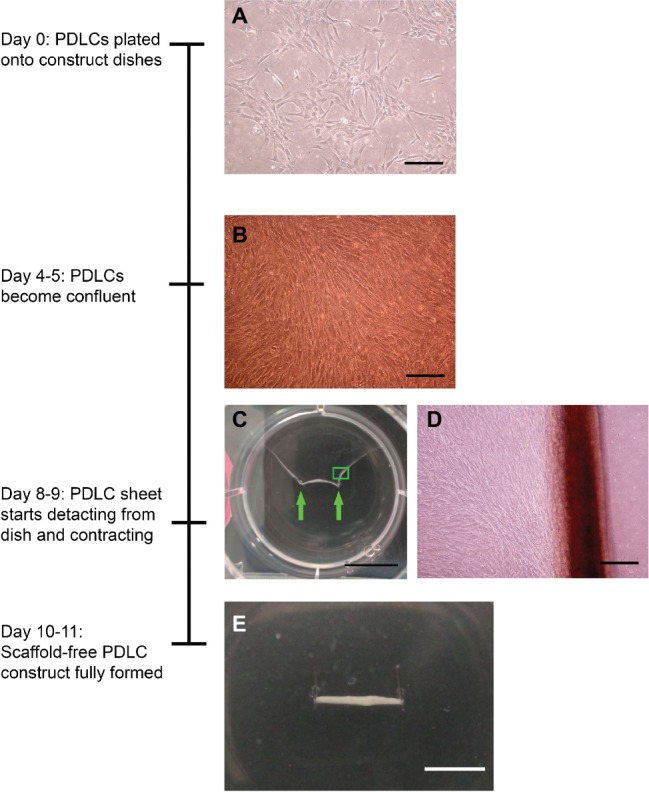
Time line of scaffold-free PDLC construct formation. **(A)** Micrograph of PDLCs after attaching to construct dishes. **(B)** Micrograph of PDLCs reaching confluence on construct dishes approximately 4–5 days following cell plating. **(C)** Image of PDLC sheet contracting from sides of well of six-well plates toward two pins placed in the center of dish; green arrows are pointing to pins. **(D)** Higher magnification micrograph of green boxed area is shown in **(C)**. **(E)** Image of final, cylindrical scaffold-free PDLC construct anchored to the dish on either end by pins. Scale bars: **(A**,**B**,**D)** = 200 μm, **(C)**: 10 mm, and **(E)**: 5 mm.

Figure 1 shows a timeline of scaffold-free PDLC construct formation. Scaffold-free 3D-engineered tissues were formed by plating PDLCs onto construct dishes. Four to five days after cell plating, the cells became confluent. The cells formed a tissue sheet that started delaminating from the substrate and rolling toward two pins in the center of the dish at 8–9 days after plating. Ten to eleven days following cell plating, the cell sheet completed rolling up to form solid, cylindrical, scaffold-free construct anchored on either end by the minutien pins. Constructs were cultured for 7 or 14 days following 3D tissue formation.

**Figure 2:**
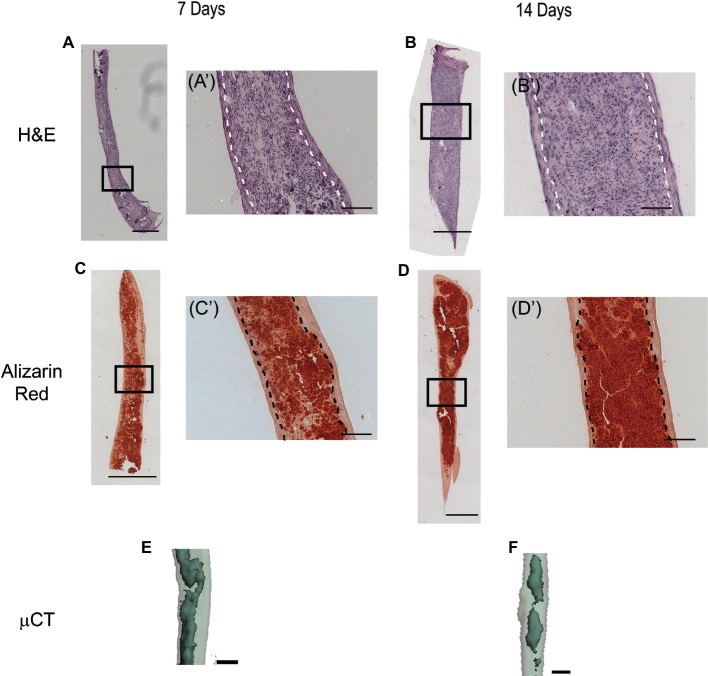
H&E, alizarin red staining, and μCT analysis of scaffold-free PDLC constructs following *in vitro* culture. **(A)** Image of H&E staining of full, longitudinal histological section of scaffold-free construct following 7 days in culture shows that the construct is solid and highly cellular. **(Aʹ)** Higher magnification image of boxed region shown in **(A)** shows that the scaffold-free constructs formed two different tissue structures, a center dense tissue with round cells and a peripheral structure containing cells and matrix elongated along the longitudinal axis of the construct; the white dotted line delineates the two structures. Similar histological features are seen in scaffold-free constructs following 14 days in culture. **(B)** H&E staining of full histological section of scaffold-free construct after 14 days in culture. **(Bʹ)** Higher magnification of boxed region shown in **(B)**; the white dotted line separates the peripheral and the core structures. **(C)** Image of alizarin red staining of full-length, longitudinal histological section of engineered tissue after 7 days in culture shows positive staining for calcium (red) indicative of mineral deposition along the length of the samples. **(Cʹ)** Higher magnification image of boxed region in **(C)** shows that positive alizarin red staining is localized to the center of the construct and a band of uncalcified tissue is on the periphery; the dashed black line separates the mineralized tissue center from the unmineralized peripheral tissue. A similar localization of mineralized tissue is seen after 14 days of culture. **(D)** Alizarin red staining of full histological section of engineered tissues after 14 days in culture. **(Dʹ)** Higher magnification image of boxed region in **(D)**; dashed black line separates peripheral uncalcified tissue from the center mineralized tissue. **(E)** Three-dimensional rendering of μCT scan of segment of engineered tissue after 7 days in culture shows mineralized tissue (dark, solid gray) localized within soft tissue (light, translucent gray). A similar 3D rendering is seen after 14 days in culture. **(F)** Three-dimensional rendering of μCT scan of segment of engineered tissue after 14 days in culture shows mineralized tissue (dark, solid gray) enclosed within a soft tissue (light, translucent gray). Scale bars: **(A–D)** = 500 μm; **(Aʹ–Dʹ**,**E**,**F)** = 100 μm.

H&E staining shows that the scaffold-free PDLC constructs are solid and cellular (Figures 2A,B). Higher magnification images show that the constructs have two distinct structures (Figures 2Aʹ,Bʹ). In the center region of the constructs, the density of cells appears higher, and the cells are more round in shape. On the periphery of the constructs, there is a clear separate structure where the cells are more elongated in shape along the longitudinal axis of the construct. The engineered tissues became highly mineralized as seen by positive alizarin red staining (Figures 2C,D). The mineral was localized to the center of the constructs, and the periphery of the constructs, the region corresponding to the elongated cell phenotype seen in H&E, completely lacks mineral (Figures 2Cʹ,Dʹ). These data are further validated by 3D μCT reconstructions of the engineered tissues showing a mineralized structure enclosed within a soft tissue (Figures 2E,F). These data show that scaffold-free tissue engineering facilitates PDLCs to self-assemble into two distinct structures one in the core of the construct and a second on the periphery. These structures are morphologically different, and furthermore, the tissue at the center of the construct becomes mineralized, whereas the peripheral tissue does not.

**Figure 3:**
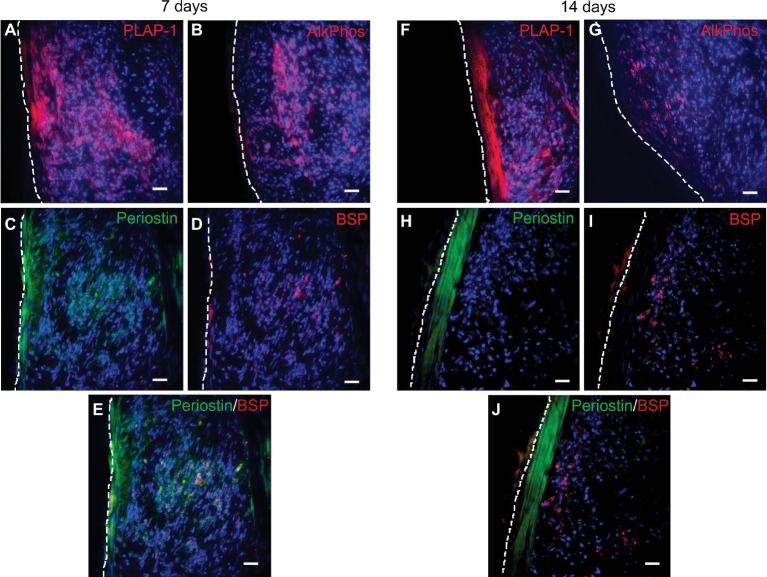
Immunofluorescent staining of scaffold-free PDLC constructs after *in vitro* culture. **(A)** PLAP-1 (red) is found expressed strongly at the periphery of the PDLC and also in the center after 7 days in culture. **(B)** ALP (red) is found expressed in the center of the scaffold-free tissue after 7 days in culture. **(C)** Periostin (green) is strongly expressed on the periphery and also found in the center of scaffold-free construct following 7 days of culture. **(D)** BSP (red) expression is found localized mainly in the center of the engineered tissue following 7 days of culture. **(E)** Merged image of **(C)** and **(D)** showing the separate localized expression of periostin (green) and BSP (red). **(F)** PLAP-1 (red) expression is found strongly associated to the periphery of the construct at 14 days in culture. **(G)** ALP (red) expression can be seen localized to the center of the engineered tissue at 14 days in culture. **(H)** Following 14 days in culture, periostin (green) is only expressed on the peripheral tissue of the scaffold-free construct. **(I)** BSP (red) expression is localized to the center of the scaffold-free construct following 14 days in culture. **(J)** Merged image of **(H)** and **(I)** showing localized expression of periostin (green) and BSP (red). In all images, DAPI was used to stain cell nuclei (blue), and white dashed line denotes the edge of the engineered tissue. Scale bars in all images = 100 μm.

Immunostaining was performed to further characterize PDLC differentiation and tissue assembly within the scaffold-free construct. At 7 days in culture following construct formation, periodontal ligament-associated protein 1 (PLAP-1) and periostin, markers of PDL, were strongly expressed on the periphery of the construct and also found in the center (Figures 3A,C). At 14 days, PLAP-1 and periostin expression were more specifically localized to only the peripheral structure of the engineered tissues (Figures 3F,H). This indicates that the peripheral PDLCs are exhibiting a periodontal ligament fibroblast phenotype. Since the localization of PLAP-1 and periostin expression becomes more specific to the periphery of the engineered tissue between 7 and 14 days, perhaps this additional time in culture facilitates tissue organization or maturation. Following both 7 and 14 days in culture, the expression of alkaline phosphatase (ALP) and bone sialoprotein (BSP), molecules characteristic of bone and cementum, was localized to the center of the construct (Figures 3B,D,G,I). This indicates that the PDLCs at the center of the engineered tissues are differentiating toward an osteo/cementoblast phenotype. Double labeling of BSP and periostin shows the distinct expression patterns of these two proteins within the engineered tissues (Figures 3E,J). These data demonstrate that when cultured as a 3D scaffold-free construct, PDLCs self-assemble such that the cells in the center of the construct differentiate into osteo/cementoblast-like cells and deposit a mineralized tissue and the peripheral PDLCs differentiate into PDL fibroblast-like cells producing a tissue resembling the periodontal ligament.

### Characterization of Scaffold-Free PDLC Constructs Following *in vivo* Implantation

**Figure 4:**
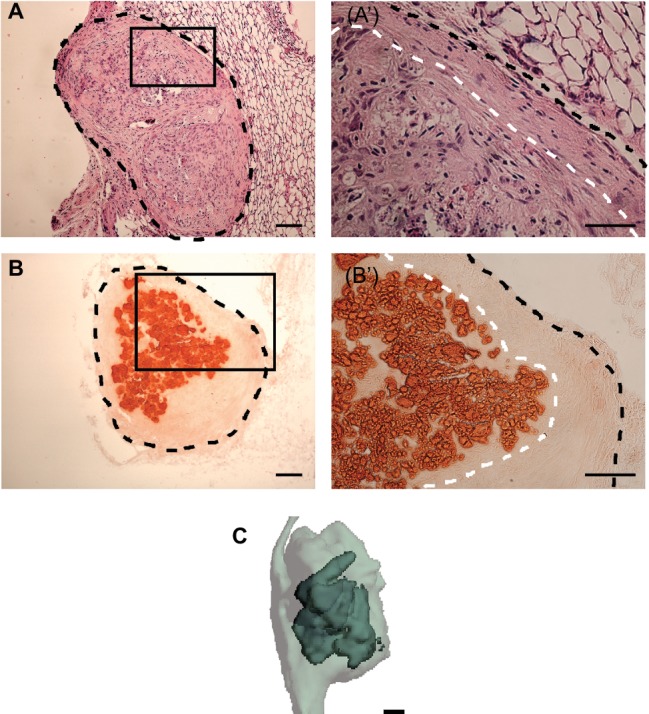
H&E, alizarin red staining, and μCT analysis of scaffold-free PDLC constructs following *in vivo* implantation. **(A)** H&E staining of histological section of engineered tissue explant following 4-week subcutaneous implantations. Black, dashed line outlines explant. **(Aʹ)** Higher magnification image of boxed region in **(A)** shows the formation of two morphologically distinct tissues on the explant, a core tissue and a separate tissue on the periphery; black, dashed line outlines the sample and white, dashed line separates the inner and outer tissue structures. **(B)** Alizarin red staining of full explant following 4-week implantation. Black, dashed line outlines full explant. **(Bʹ)** Higher magnification image of boxed region in **(B)** shows that the core of the explant is stained positively for alizarin red while the periphery does not. Black, dashed line outlines the explant and the white, dashed line delineates the mineralized core tissue from the peripheral unmineralized tissue. **(C)** Three-dimensional rendering of μCT scan of explant shows mineralized tissue (dark, solid gray) localized within soft tissue (light, translucent gray). Scale bars: **(A**,**B**,**B**ʹ,**C)** = 100 μm, **(Aʹ)** = 50 μm.

Following 7 days of *in vitro* culture, scaffold-free PDLC constructs were implanted subcutaneously in immunocompromised mice to assess the effect of the *in vivo* environment on the engineered tissues. While implanted, the scaffold-free engineered tissues remodeled from the cylindrical shape seen *in vitro* to more of a spherical shape. Following a 4-week implantation, H&E staining showed that the PDLC constructs maintained the tissue morphology seen after *in vitro* culture with a dense, cellular core tissue and a morphologically distinct peripheral tissue comprising elongated cells and matrix (Figures 4A,Aʹ). Furthermore, the center tissue within the explants was mineralized as seen by positive alizarin red staining (Figures 4B,Bʹ), and the peripheral tissue remained uncalcified. μCT verified that the explants comprised a mineralized core localized within a soft tissue (Figure 4C).

**Figure 5:**
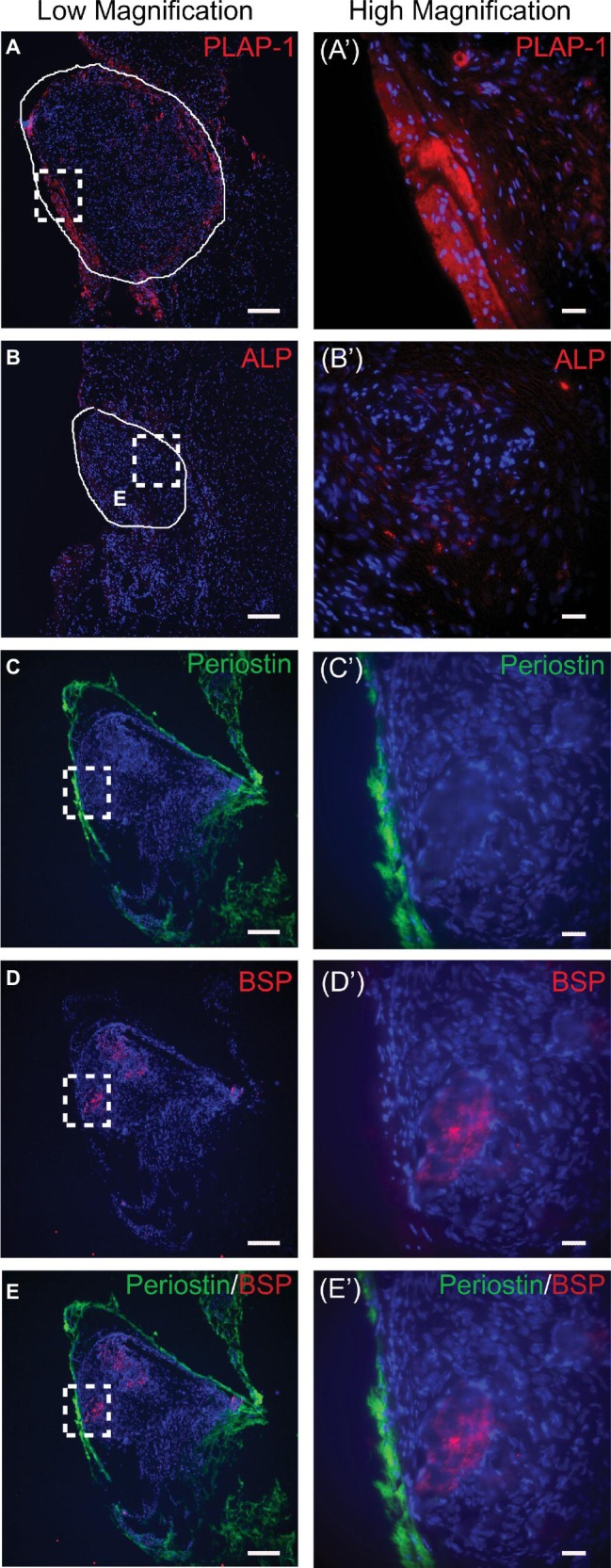
Immunofluorescent staining of scaffold-free PDLC constructs after 4-week *in vivo* implantation. **(A)** PLAP-1 (red) is expressed strongly on the periphery of the scaffold-free constructs; solid white line outlines engineered tissue. **(Aʹ)** Higher magnification image of dashed boxed region shown in **(A)**. **(B)** Faint ALP (red) expression can be seen localized in the center of the scaffold-free constructs after implantation; solid white line outlines construct. **(Bʹ)** Higher magnification image of dashed boxed region shown in **(B)**. **(C)** Periostin (green) is expressed on the periphery tissue of scaffold-free construct following implantation. **(Cʹ)** Higher magnification image of boxed region in **(C)**. **(D)** BSP (red) is expressed in the center tissue of scaffold-free construct following implantation. **(Dʹ)** Higher magnification image of boxed region in **(D)**. **(E)** Merged image of **(C)** and **(D)** further validates that periostin (green) is expressed on periphery tissue and BSP (red) is expressed in the center tissue of engineered construct. **(Eʹ)** Higher magnification of boxed region shown in **(E)**. In all images, DAPI was used to stain cell nuclei (blue). Scale bars: **(A**–**E)** = 100 μm; **(Aʹ**–**Eʹ)** = 50 μm.

Immunostaining of the explants showed that PLAP-1 (Figures 5A,Aʹ) and periostin (Figures 5C,Cʹ) were strongly expressed only on the periphery of the construct. ALP (Figures 5B,Bʹ) and BSP (Figures 5D,Dʹ) expressions were localized in the center of the constructs, in a similar region of the mineral deposition. After *in vivo* implantation, only a low level of ALP expression is seen. ALP is an early marker of osteo/cementogenic cell differentiation involved at early stages of mineral deposition. It is therefore not surprising that strong ALP expression is not seen after *in vivo* implantation; since at this point, the engineered tissues may be developing into a more mature phenotype. Double labeling of periostin and BSP shows that there is no overlap in signal further supporting the formation of two distinct tissues (Figures 5E,Eʹ). This indicates that after *in vivo* implantation the PDLC-engineered tissues were able to maintain the tissue organization assembled during *in vitro* culture with an osteo/cementogenic center enclosed within a PDL-like tissue.

## Discussion

An effective therapy to regenerate the periodontium will require directing cells to rebuild the various periodontal tissues in an organized manner. Understanding the cellular mechanisms facilitating periodontal tissue patterning is therefore critical. In this study, unique 3D scaffold-free constructs were formed from PDLCs that facilitated the cells to self-assemble to form an osteo/cementogenic core with a periodontal ligament-like periphery, similar to what is seen naturally on the surface of a tooth root. The formation of these organized structures is seen after *in vitro* culture and is maintained after *in vivo* implantation. These engineered constructs could be used as functional grafts to rebuild periodontal defects or used as a controllable model system to study periodontal ligament cell fate decisions and the complex tissue assembly involved in rebuilding the periodontium.

Cell sheet engineering is another form of scaffold-free tissue engineering that has been explored as a method to regenerate the periodontium (Hasegawa et al., 2005; Iwata et al., 2009; Zhou et al., 2012; Chen et al., 2016). The 3D scaffold-free constructs that we have formed and characterized in this present work differ from the cell sheets that have been previously reported in multiple significant ways. Cell sheets are formed by culturing cells to confluence and allowing the cells to produce their endogenous extracellular matrix to form a layer of tissue that can be separated from the substrate. In our system, the cells first form cell sheets and then are further cultured so that the cell sheet contracts and rolls up into a robust 3D cylindrical tissue. The shape and dimensionality of our constructs therefore are very different what is seen in cell sheets. Furthermore, our scaffold-free system facilitated cells within the same engineered tissue to self-assemble to form both osteo/cementogenic and PDL-like tissues, a feature that has not yet been seen in cell sheets. The previously reported research on the use of scaffold-free cell sheets, however, has established the safety and feasibility of utilizing cellular, scaffold-free engineered tissues for regenerating periodontal tissues. Researchers have found that PDLC sheets aid in periodontal regeneration in both small and large animal defect models (Hasegawa et al., 2005; Iwata et al., 2009; Zhou et al., 2012). Furthermore, studies have been performed to assess the use of autologous PDLC sheets for periodontal regeneration in humans (Chen et al., 2016). This research validates the safety and feasibility of translating scaffold-free cellular tissue engineering therapies into humans.

In this study, scaffold-free PDLC constructs were shown to organize into two morphologically distinct tissues, an outer fibrous PDL-like structure and an inner mineralized tissue. The formation of a PDL-like structure on the periphery of the engineered tissues was determined in part by the positive expression of PLAP-1 and periostin. PLAP-1, also known as asporin, is a member of the small leucine-rich proteoglycan (SLRP) family and is preferentially expressed in the periodontal ligament. PLAP-1 has been shown to prevent osteo/cementogenic differentiation of PDLCs and the mineralization of PDL tissue by inhibiting the Smad signaling pathway (Yamada et al., 2001, 2007; Ueda et al., 2016). Periostin is an extracellular matrix protein found in a number of tissues, including the PDL, periosteum, cardiac valves, alveolar wall, and cancer-associated stroma, and has multiple functions including involvement in extracellular matrix assembly and in cell adhesion (Horiuchi et al., 1999; Kii and Ito, 2017). Within the PDL, periostin has been shown to have a role in tissue remodeling in response to mechanical loading, and periostin-deficient mice develop an early onset periodontal disease phenotype (Rios et al., 2005, 2008). This factor is therefore critical for proper PDL homeostasis. It could be argued that since periostin is also expressed naturally in the periosteum, the peripheral tissues on the scaffold-free PDL constructs could be reminiscent of a periosteal structure rather than a PDL-like tissue; however, the peripheral tissue on these engineered tissues is structurally dissimilar to a periosteum. In our previous studies, we have reported the formation of a periosteum-like structure on scaffold-free tissues engineered using bone marrow stromal cells (Syed-Picard et al., 2009, 2014). In these studies, the engineered periosteum, in addition to exhibiting biochemical characteristics of a periosteum, also displayed morphological features of a natural periosteum comprising both the fibrous and cambium layers of a true periosteum. These types of structural landmarks characteristic of a periosteum were not seen in the scaffold-free PDL constructs reported here. Therefore, in this current study, the localized expression of periostin and PLAP-1 on the periphery of the scaffold-free PDL constructs in addition to the morphology and uncalcified nature of the peripheral tissue is indicative of PDL tissue formation.

Natural periodontal ligament has a characteristic morphology consisting of cells and ECM fibers oriented perpendicular to the surface of the cementum and the alveolar bone. These morphological features are a result of the mechanical forces placed on the PDL *in situ* during tooth development, eruption, and function (Nanci and Ten Cate, 2013). Unlike true PDL, the PDL-like tissue in our engineered constructs appears to be aligned parallel to the osteo/cementogenic core tissue. We speculate that this result is because the engineered constructs are being generated and cultured without the mechanical stimuli present in the natural tooth socket. Potentially, subjecting our engineered constructs to the mechanical environment of natural PDL could stimulate the cells and ECM in the PDL-like tissue to rearrange to more closely emulate what is seen in natural PDL. These engineered constructs could be a useful model system to study the effect of mechanical loading on PDL cells and ECM.

Scaffold-free tissue engineering of PDLC resulted in the formation of an osteo/cementogenic tissue localized in the center of the construct. Cementum is biochemically very similar to bone and dentin; therefore, from compositional perspective, it is very difficult to distinguish these tissues. The extracellular matrices of all of these mineralized tissues comprise predominately type I collagen, and these tissues all contain similar enzymes and non-collagenous extracellular matrix proteins, including ALP and BSP, which are involved in mineralization processes (Nanci and Ten Cate, 2013). In this study, we characterized the tissue located at the center of our construct based on its mineralized nature and the positive expression of ALP and BSP; however, these characteristics alone are not sufficient to truly define the tissue at the core of construct as cementum instead of bone. Due to the compositional similarities between bone and cementum, some have argued that cementoblasts are simply positional osteoblasts, and cementum is a specialized type of bone located on the surface of the tooth between the dentin and the PDL (Foster, 2012). Based on this type of positional identity, the mineralized tissue localized at the core of the scaffold-free engineered tissues formed in this study can be characterized as cementum-like since it is located within a PDL-like tissue.

Here, we have established a scaffold-free engineered tissue that could be used as a controllable model system to study the assembly of periodontal tissues and have provided the foundational characterization of these engineered constructs. However, tissue-engineered model systems do not fully emulate native structures since they are not generated in a setting that fully encompasses the complex milieu of chemical and mechanical signals provided in an *in situ* environment. In our current study, we implanted our engineered PDLC constructs subcutaneously in mice. In future studies, it would of interest to evaluate these scaffold-free constructs after implantation either into the tooth socket or in the periodontal space between dentin and alveolar bone. In addition, in this study, the PDLC constructs were formed in static culture, and it is established that mechanical stimuli are critical for periodontal tissue organization and morphology. It is, therefore, critical to further assess these engineered tissues following mechanical stimulation to assess if these engineered tissues respond to external forces in a similar manner to native tissues.

We have formed scaffold-free engineered tissues using PDLCs that self-assembled into an organized PDL-cementum-like complex. These engineered tissues have feasible regenerative potential for treating periodontal disease, and furthermore, these engineered constructs could be used as unique and powerful model system to study the mechanisms driving PDLCs to properly assemble a cementum-PDL complex.

## Ethics Statement

All animal procedures were approved by the University of Pittsburgh Institute of Animal Care and Use Committee.

## Author Contributions

AB was involved in study design, performed experiments, and collected and analyzed data. KR and MA performed experiments and collected data. FS-P was involved in study design, data analysis and interpretation, and manuscript preparation.

### Conflict of Interest Statement

The authors declare that the research was conducted in the absence of any commercial or financial relationships that could be construed as a potential conflict of interest.
